# Characterizing Learners’ Complex Attentional States During Online Multimedia Learning Using Eye-tracking, Egocentric Camera, Webcam, and Retrospective recalls

**DOI:** 10.1145/3649902.3653939

**Published:** 2024-06-04

**Authors:** Prasanth Chandran, Yifeng Huang, Jeremy Munsell, Brian Howatt, Brayden Wallace, Lindsey Wilson, Sidney D’Mello, Minh Hoai, N. Sanjay Rebello, Lester C Loschky

**Affiliations:** Department of Psychological Sciences, Kansas State University, Manhattan, Kansas, United States; Computer Science Department, Stony Brook University, Stony Brook, New York, United States; Dept. of Physics & Astronomy, Purdue University, West Lafayette, Indiana, United States; Department of Psychological Sciences, Kansas State University, Manhattan, Kansas, United States; Department of Psychological Sciences, Kansas State University, Manhattan, Kansas, United States; Department of Psychological Sciences, Kansas State University, Manhattan, Kansas, United States; University of Colorado Boulder, Boulder, Colorado, United States; Computer Science Department, Stony Brook University, Stony Brook, New York, United States; Dept. of Physics & Astronomy, Purdue University, West Lafayette, Indiana, United States; Department of Psychological Sciences, Kansas State University, Manhattan, Kansas, United States

**Keywords:** Multimodal data, Eye-tracking, Online learning, Attentional States

## Abstract

As online learning becomes increasingly ubiquitous, a key challenge is maintaining learners’ sustained attention. Using eye-tracking, together with observing and interviewing learners, we can characterize both 1) whether they are looking at their learning materials, and 2) whether they are thinking about them. Critically, eye-tracking only speaks to the first distinction, not the second. To overcome this limitation, we supplemented eye-tracking with an egocentric camera, a webcam, a retrospective recall, and mind-wandering probes to capture a 2×2 matrix of attentional/cognitive states. We then categorized N=101 learners’ attentional/cognitive states while they completed a multimedia physics module. This meets two goals: 1) allowing basic research to understand the relationship between attentional/cognitive states and behavioral outcomes; and 2) facilitating applied research by generating rich ground truth for future use in training machine learning to categorize this 2×2 set of attentional states, for which eye-tracking is necessary, but not sufficient.

## 1 INTRODUCTION

Online learning is here to stay. But the Achilles’ heel of online learning is its difficulty in gauging learners’ attention. This is because attention is critically important for learning [Wong et al., 2022, Erickson et al., 2015, Reynolds and Shirey, 1988, Hidi, 1995]. Thus, effective online instruction should maintain learners’ attention, just as good teachers work to do in the classroom [Wolff et al., 2016, Goldberg et al., 2021]. Because most online instruction does not measure learners’ level of attention, a good starting point, if possible, is to use eye tracking measures, which are among the best online attention metrics [Duchowski, 2017, Findlay and Gilchrist, 2003, Holmqvist and Andersson, 2018]. Still, users of eye tracking must avoid oversimplification concerning the meaning of looking at the screen learning materials. Specifically, there is a complex interplay between learners’ gaze, their attention, and learning. First, learners’ attentional states are not always aligned with their external gaze, but can instead be oriented internally to either on- or off-task thoughts (e.g., plans for the evening) [Smallwood and Schooler, 2006, Schooler et al., 2011]. Second, just because a learner’s gaze has left their learning materials, does not mean they are off-task. They could be looking elsewhere to take notes, or to think deeply about what they just read–both of which can be valuable to learning. These considerations call for a broader conceptualization.

### 1.1 Theoretical Framework & Goals of Study

**Figure 1:**
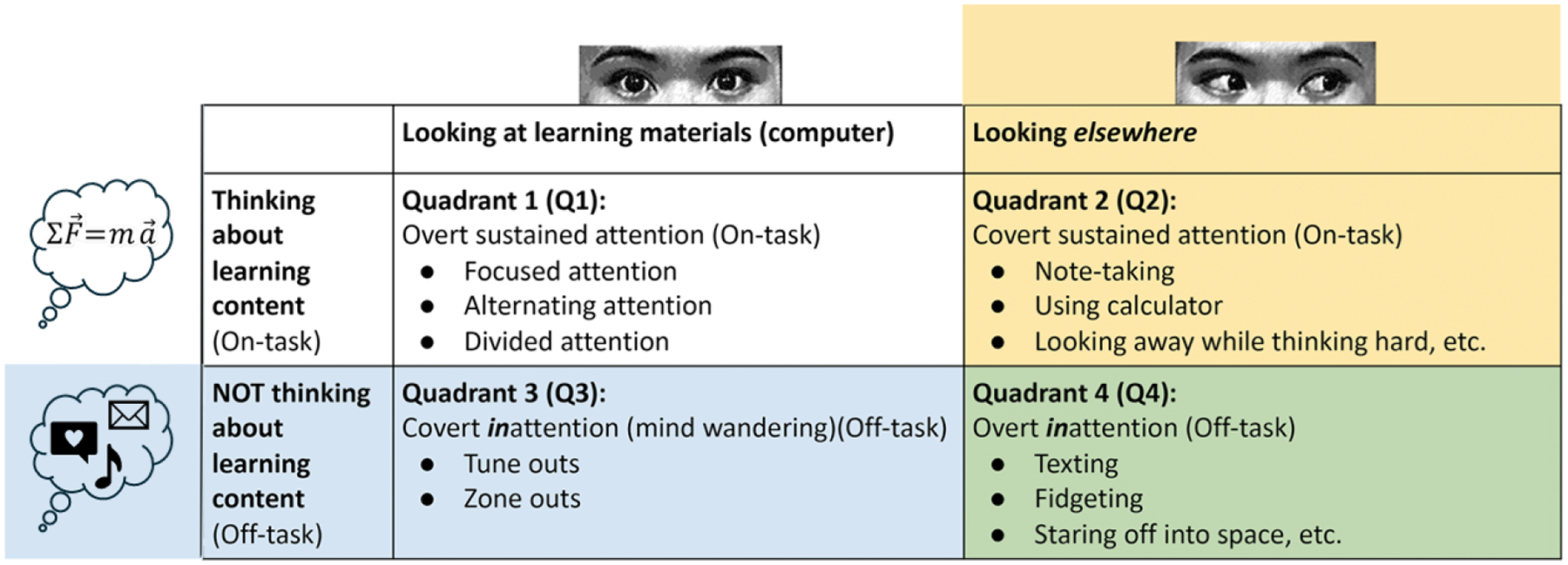
A 2×2 Matrix of Learners’ Attentional & Cognitive States in Online Learning (lightly revised) [D’Mello, 2016]).

The above distinctions can be mapped onto a 2×2 matrix, shown in Figure 1, in which learners can either look at the learning materials on the computer screen (left column) or look elsewhere (right column), and their attention can either focus on the learning content (top row) or on something else (bottom row) [D’Mello, 2016]. The goal of the present study is to map the 2×2 attentional/cognitive states of learners as they complete a multimedia instructional module on introductory physics. Information about online learners’ attentional/cognitive states can be used by instructors, or instructional designers, to revise their learning materials to optimally engage their learners (e.g., switching from lecture to group work) [Goldberg et al., 2021].

A long-term goal of this project is to use the ground truth on these 2×2 states measured in the current study to train intelligent attention-aware software using machine learning to track online learners’ attentional/cognitive states in real-time. Such software can potentially be incorporated into human-computer interfaces to ask learners if they need a break, to restore attentional resources [Ginns et al., 2023], or prompt them with a short quiz, to estimate if reviewing the recently presented materials would help their learning [Mills et al., 2020].

### 1.2 Proposed Approach: Additional Measures to Produce Ground Truth for the 2×2 Matrix

#### 1.2.1 Novelty & Research Questions.

To date, we have found no significant research that directly applies the full 2×2 matrix in Figure 1 to characterize learners’ moment-to-moment attentional/cognitive states during online learning. To do this, we start with eye tracking, but supplement it to overcome the two limits noted earlier. If eye tracking tells us that the learner is looking at the learning materials (left column in Fig 1), we supplement that information by using periodic mind-wandering probes (i.e. online self-reports) to ask the learner if they are indeed thinking about the learning content (top row) or not (bottom row) [Robison et al., 2019]. Conversely, if the eye tracking tells us that the learner is looking elsewhere (right column in Fig 1), we supplement that with information from an egocentric camera that shows what they are looking at, and a later retrospective recall protocol to ask if they were thinking about the learning materials. By supplementing eye tracking in this way, we can measure whether learners are on-task (top-row) versus off-task (bottom-row in Fig 1) [Goldberg et al., 2019, Baker et al., 2020]. The novel contribution of the current study is to have simultaneously investigated all four quadrants of the 2×2 matrix shown in Figure 1, and investigate its relationship to learning. Thus, we address the following research questions:

RQ1: Can one account for online learners’ attentional and cognitive states over the entire period of an extensive lesson using our 2×2 matrix?

RQ2: To what extent does the fraction of time spent by learners in various 2×2 attentional/cognitive predict their learning outcomes?

## 2 METHOD

### 2.1 Participants

A total of 101 participants (NFemale = 53, NMale = 48) were recruited through a daily online university-wide publication (age: M = 28.08, SD = 7.82). Participants were relatively ethnically diverse (available race demographics based on 79 participants: 40.51% Asian, 36.70% White, 10.13% Black or African-American, 6.33% Middle Eastern or North African, 3.80% Hispanic, Latino or Spanish and 2.53% other). Participants gave informed consent for their participation, and the study was authorized by the Institutional Review Board. Participants were paid $60 for their participation, which took, on average, 3 hours.

### 2.2 Stimuli (Learning Module)

The stimuli in the study were a multi-modal learning module on Newton’s Second Law. The module consisted of four major sections. It began with two short videos (7 min 35 sec and 5 min 43 sec long) on how to write and solve Newton’s Second Law equation using a force diagram. Then, there was a Review page summarizing the formulas and diagrams covered during the videos. Finally, there was a practice question to apply the concepts learned during the module. After answering the practice question participants could also watch a feedback video (2 min 41 secs long) showing them how to solve the practice question. Participants had the option to pause, rewind, or skip the videos whenever they chose, and they also had the option to go back to earlier sections of the module.

### 2.3 Instruments

**Figure 2:**
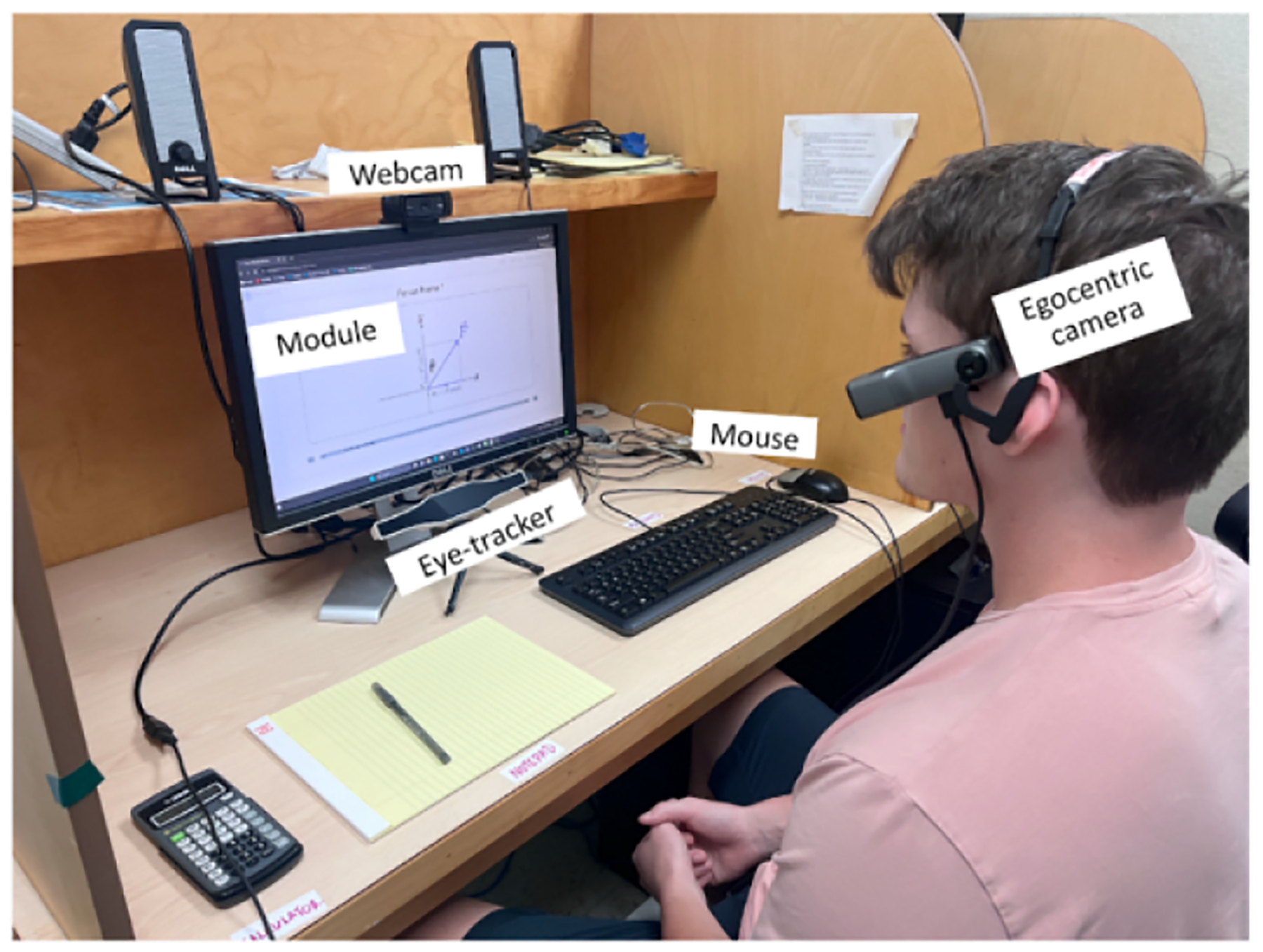
Instruments used for collecting multi-modal data in the study.

As shown in Figure 2, above the computer screen a webcam provided video of the learner’s face, upper body, and sometimes their hands, at a sampling rate of 60 Hz. Beneath the computer screen a commercial grade eye tracker (GazePoint3) measured where on the computer screen the learner was looking, also sampled at 60 Hz. On the desktop, there was a computer mouse and keyboard for the learner to interact with the module, again sampled at 60 Hz. Learners wore a 60 Hz egocentric camera, which captured what the learner was looking at, including their hands or other items (e.g., cellphone) extending beyond the view of either the webcam or eyetracker. Participants were also provided with a notepad, pen, and calculator to take notes or solve problems. To allow for the possibility of participants going off-task by using their cellphones (a common real-world distraction), they were told that they could use their cellphones during the module, if they chose to.

### 2.4 Procedure

Our study began with a 26-item pre-test over the to-be learned content, followed by a multimodal learning module on Newton’s second law. We then gave participants a retrospective recall interview, and then the post-test (identical to the pre-test). Roughly one week later, participants took a final retention test (identical to the pre- and post-tests) proctored on Zoom. Approximately 4–8 months later, we invited our participants to fill out a demographic questionnaire (from the 2020 US Census), and a 16-item socially desirable responding questionnaire.

### 2.5 Data Post-processing and Categorization

#### 2.5.1 Coding of Learners Moment-by-Moment Attentional States into the 2×2 Matrix.

Figure 1 shows our 2×2 matrix of overt attention and cognitive states. We used the eye tracker to automatically distinguish between the columns (looking at the learning materials vs looking elsewhere), then used self-reported mind wandering (via online thought probes) and retrospective cued recall to assess the content of thoughts.

#### 2.5.2 Eye tracking.

We first distinguished between looking at the learning materials on the computer screen versus elsewhere. The learning materials on the computer screen were defined using a predetermined rectangular area of interest showing the module video. Using an eye tracker, we estimated whether a participant’s overt attention was on the learning materials, namely Column 1 (white and blue in Figure 1). Otherwise, their overt attention was elsewhere, namely Column 2 (yellow and green in Figure 1).

#### 2.5.3 Mind Wandering Probes.

We next focused only on those cases in which the participant *was* looking at the learning materials, represented by the left column of Figure 1 (shown in white and blue). We then distinguished whether the participant was *thinking about* the learning materials (i.e., were they mind wandering?) using probes [Weinstein, 2017]. Every 120–150 seconds [Mills et al., 2020] (randomly jittered), we replaced the on-screen learning materials with a probe asking, ”Were you zoning out?” and participants responded by key press, either ”Y” for ”yes” or ”N” for ”no,” which removed the probe screen. If the participant responded ”No” (*not* zoned out), we categorized them as thinking about the learning content, namely the top row of Figure 1 (on-task). Here, learners were both looking at the learning content, and thinking about it, namely the top-left quadrant (shown in white in Figure 1). Otherwise, for a ”Yes” (i.e., zoned out) response to the mind-wandering probe, we categorized them as *not* thinking about the learning content (i.e., off-task) for the 15 secs before the probe [Krasich et al., 2018, Faber et al., 2020]. In this case, they were in the bottom-left quadrant (shown in blue in Figure 1). This distinction between being on-task versus mind-wandering, while looking at the learning materials, has been extensively studied in the mindless-reading and mind-wandering literatures [for reviews, see Wong et al., 2022, Mills et al., 2020, Faber et al., 2020].

#### 2.5.4 Retrospective cued recall using screen capture, webcam, and egocentric camera.

We next focused on cases in which the participant was *not* looking at the learning materials (using eye tracking), to determine whether they were *thinking about* the content (i.e., on- vs. off-task). Note that, in these cases, eye tracking only tells us that the participant was not looking at the learning materials (i.e., in Figure 1, the right column, in yellow and green). To distinguish whether learners were on- versus off-task, we again used mind-wandering probes, but we added a retrospective recall interview to gain richer information. Specifically, any time a participant looked away from the learning content for 2 seconds or longer, our software inserted a flag in the data stream. Once the participant completed the learning module, we synchronized their eye tracking, egocentric camera, webcam, screen content, and mouse data. This data was segmented into 15 second periods, each beginning with the eye tracking flag. Our Research Assistants gave the retrospective recall interviews, and showed the participant three data streams for each 15 second period: 1) video of the learning material on-screen, 2) webcam images of the participant, and 3) egocentric camera images. Based on these data sources, we asked participants to report if they were engaged in various on- or off-task behaviors.

**Figure 3:**
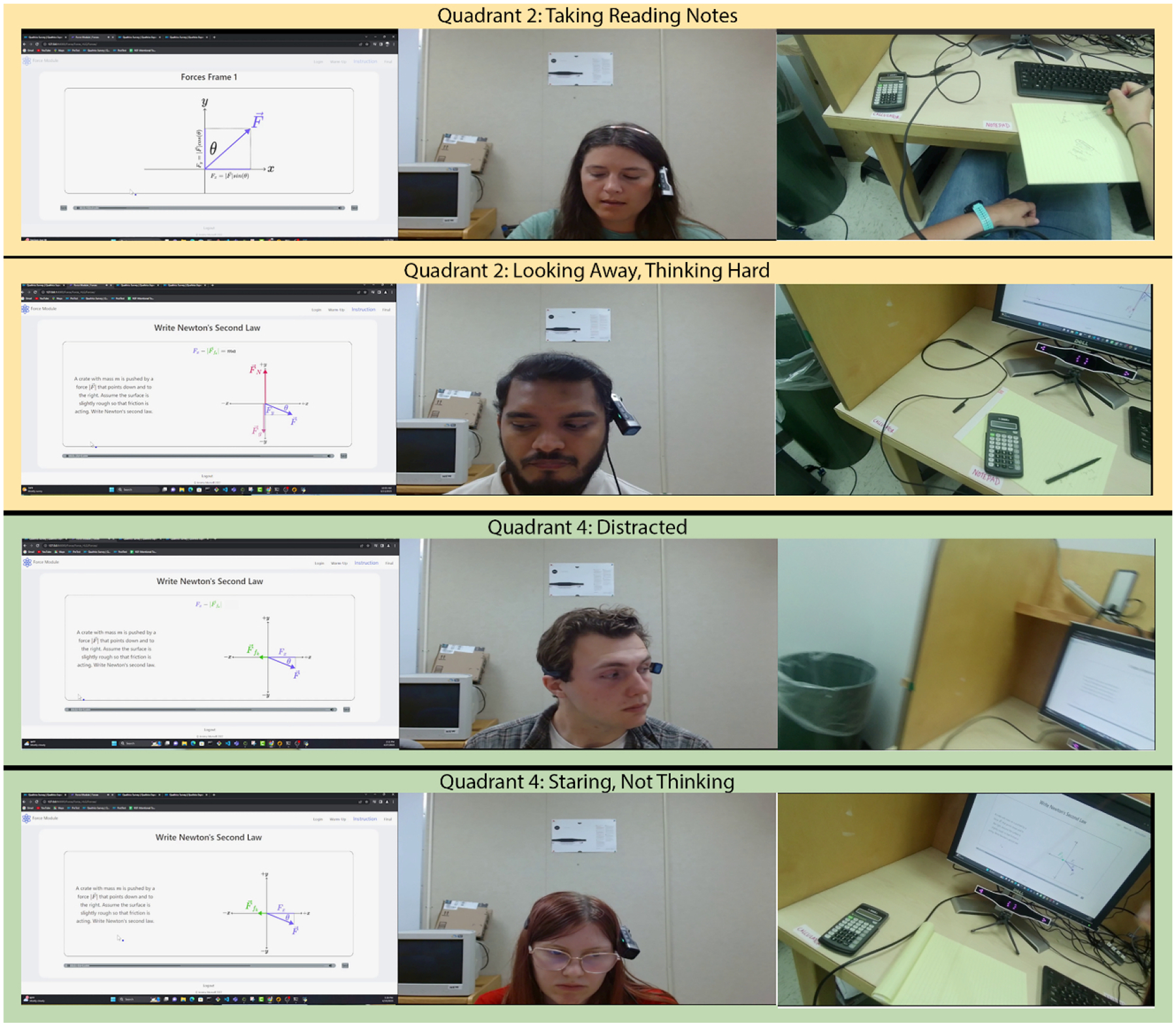
Examples of multi-modal data (from left: screen capture, webcam, and egocentric camera view) used in the retrospective recall interview for categorizing on- and off-task behaviors while looking away from the learning materials.

Figure 3 illustrates the above process. In the top row, the webcam shows the learner looking away from the screen, while the egocentric camera shows that they appeared to be taking notes. The Research Assistant asked the participant to confirm that, and coded them as being in the top-right quadrant of the 2×2 matrix. Importantly, since note-taking has been shown to promote learning [Kobayashi, 2006, Jansen et al., 2017], this is a good case of a participant *looking away from the learning materials, while beneficially thinking about them*.

In the second row of Figure 3, the webcam again shows a learner who is not looking at the learning materials, but their egocentric camera does not show them doing any particular activity. Thus, the Research Assistant asked if they were thinking about the learning materials, and, here, the participant answered ”Yes.” Thus, this was coded as ”looking away, thinking hard.” Specifically, this is a case of *non-visually guided eye movements* aka *gaze aversion* [Servais et al., 2023, Ehrlichman and Micic, 2012] which people commonly do when retrieving information from long-term memory [Servais et al., 2023, Ehrlichman and Micic, 2012], or during cognitively demanding tasks like mental math [Epelboim and Suppes, 2001].

In the bottom two rows of Figure 3, the webcam shows participants looking away from the learning materials, while the egocentric camera shows no evidence of on-task behaviors. In both cases, when asked by the Research Assistant, the participants reported not thinking about the learning materials. In row 3, they reported being distracted by someone walking by. In row 4, the participant reported being zoned out.

In rare cases, the eye tracker *lost track* of *the* participant’s eyes, indicating not looking at the learning materials, but the webcam and egocentric camera indicated that they *were* looking on screen. In these cases, we asked the participant 1) if they were looking at the materials, and 2) if they were thinking about them, and categorized them accordingly.

Retrospective recall responses were further re-coded in detail by two trained coders (*cohen’s k* = 0.68 indicating adequate reliability) to improve the data quality by: 1) differentiating multiple subcategories in the 2×2 matrix, 2) fixing errors in initial coding, and 3) recategorizing behaviors not fitting in the 2×2 matrix as “uncategorized”.

## 3 RESULTS & DISCUSSION

### 3.1 Attentional States

To answer our first research question, we were able to completely categorize participant’s attentional/cognitive states within the 2×2 matrix, within a one second level of resolution during the module. Based on this categorization, we can now calculate the proportion of time spent in each of the four attentional and cognitive states in our 2×2 matrix. We only considered data from 98 participants, since three lacked complete eye-tracking data. Our participants spent most of their total time (75.8%) looking at the learning materials and thinking about them (Q1: On-materials On-task), followed by a moderate amount of time (18.8%) looking elsewhere, but thinking about the learning content (Q2: Off-materials On-task).

We estimated that participants spent 2.02% of their total time in the module looking at the materials, but mind wandering (Q3: On-materials Off-task). For this estimate, we assumed they had mind wandered for the 15 seconds prior to the probe, based on prior such estimates using eye movement metrics associated with mind wandering [Krasich et al., 2018, Faber et al., 2020]. However, this time window for mind-wandering depends on stimulus type [Mills et al., 2020], hence future analyses will refine the interval of mind-wandering for our module by further analyzing eye movement metrics prior to “Yes” responses. Although our 2% of the time estimate seems very small, our participants did respond ”Yes” to 23% (95% CI = [18.20, 27.75]) of the 7.15 mind wandering probes presented to each participant on average (*Median* = 7, *SD* = 1.48). While this rate is lower than the 31.74% mean ”Yes” rate found in a meta-analysis of 71 educational studies, it is well within the range of 9–53% they reported [Wong et al., 2022].

Our participants similarly reported being off-task while looking elsewhere only 2.2% of the time (Q4: Off-materials Off-task). Thus, participants spent the vast majority of their time (94.6%) on-task, with only 3.9% of their time spent off-task (and the remaining 1.5% of participants’ time uncategorized). The low proportion of time spent off-task may be explainable in terms of participants being aware of their attention being monitored (i.e., by webcam, eye tracker, and ego-centric camera–the Research Assistant was in another room once each participant was set up).

### 3.2 Learning

We ran a multilevel logistic regression predicting each participants’ probability of answering each question item correctly in the pre-test (*M* = 0.26, *SE* = 0.05), post-test (*M* = 0.48, *SE* = 0.07) and retention-test scores (*M* = 0.45, *SE* = 0.07). Planned comparisons using a Tukey HSD test revealed that Post-test and retention scores were significantly higher than pre-test scores (post vs pre: *Z* = 14.35, *p* < .001; retention vs pre: *Z* = −12.58, *p* < .001), showing that the module contributed to significant learning. Interestingly, there was no significant difference between their post-test and retention scores (*Z* = 1.53, *p* = .27), indicating they retained what they learned even after one week. Participants and questions were included as random intercepts in the above model.

### 3.3 Attentional states and learning

**Figure 4:**
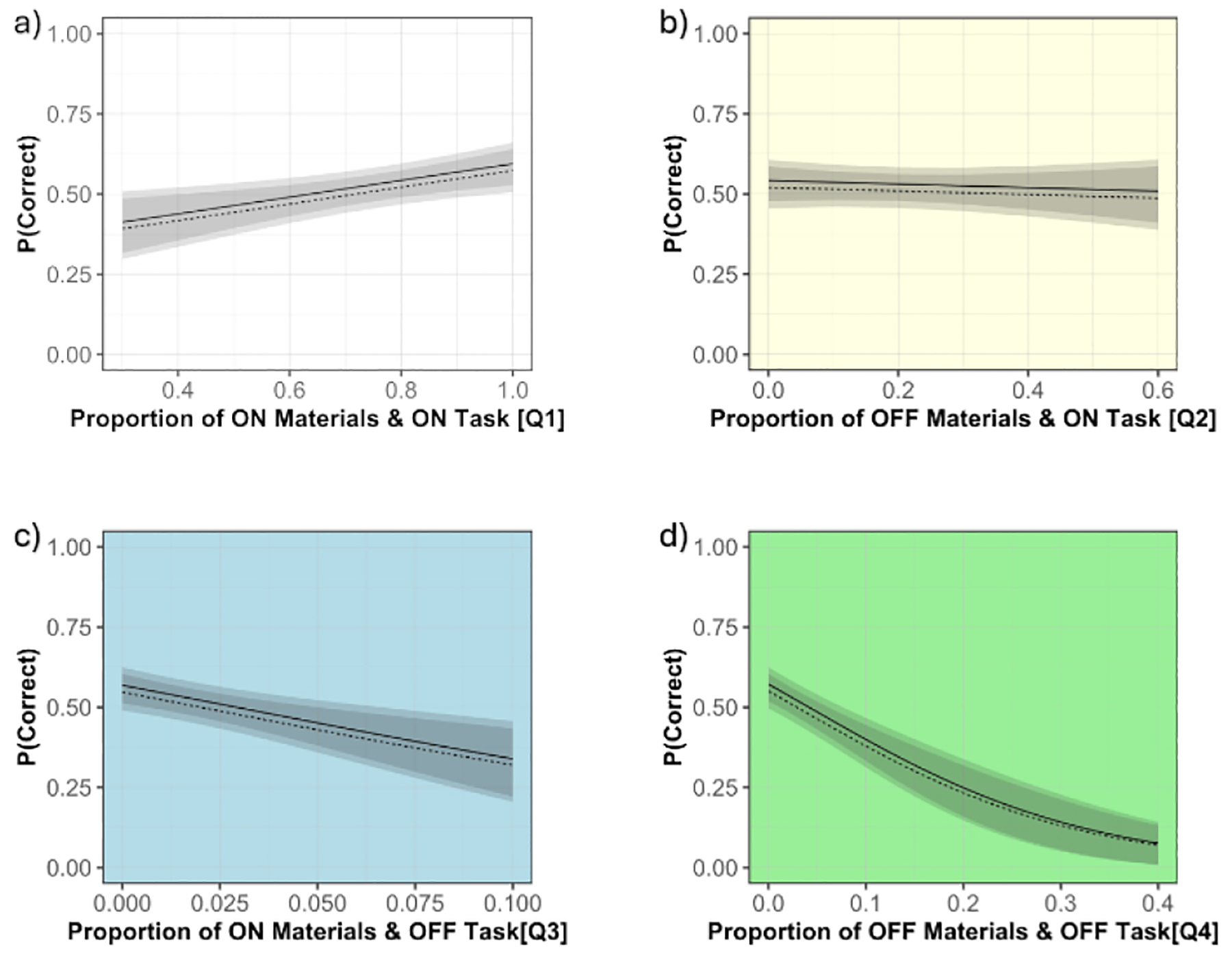
(a-d) These show relationships between time spent in each of the four attentional states and learning (i.e., for an average pre-test score). The solid line represents post-test scores, and the dotted line represents retention scores.

Our second research question was, to what extent does the fraction of time spent by learners in various 2×2 attentional/cognitive states correlate with their learning? This also serves to further test the validity of our four quadrant classification of attentional states. Thus, we tested if the proportion of time spent in each state predicted participants’ learning. We found that the attentional states were highly intercorrelated. Thus, we computed a separate logistic regression for each attentional state to predict correctness (1 or 0) of each item on the post- and retention-tests based on: 1) time spent in the corresponding attentional state, and 2) pre-test scores. Participants and questions were included as random intercepts. Both post- and retention-tests were similarly affected by 1) the proportion of time spent in each attentional state and 2) pre-test scores. Thus, we only report the effect of attentional states on post-test scores. Time spent looking at the materials and on-task (Q1) showed a non-significant positive effect on learning (*B* = *1.05*, *p* = .154)(see Figure 4a). However, time spent looking elsewhere but on-task (Q2) showed no effect on learning (*B* = *−0.22*, *p* = .784)(see Figure 4b). Conversely, for time spent looking elsewhere and off task (Q4), we found a significant negative effect on learning (*B* = −6.96, *p* = .004), and likewise but marginally significant for looking at materials, but mind wandering (Q3) (*B* = −9.42, *p* = .0989)(see Figures 4c and 4d respectively).

It is interesting that the strongest impacts on learning were the negative effects for time spent off-task. The lack of significant positive effects for time spent on-task, both when looking at the materials (in Q1) and when looking elsewhere (in Q2) may be because these behavioral categories are heterogeneous. For example, educational research has identified numerous on-task generative behaviors shown to increase learning, but which require more effort than looking at the learning materials and thinking about them (e.g., summarizing, self-explaining, enacting, etc.) [Fiorella and Mayer, 2015]. More granular analyses of subcategories of Quadrant 2 (Off-materials On-task) may reveal which on-task behaviors when looking elsewhere, such as note-taking [Kobayashi, 2006, Jansen et al., 2017], or gaze aversion while thinking deeply [Epelboim and Suppes, 2001], were most associated with learning in our study. Likewise, simply looking at learning materials and thinking about them (Q1) does not distinguish whether one is attending to thematically relevant information, associated with learning, or thematically irrelevant information, which is not [Madsen et al., 2012, Rouinfar et al., 2014]. Thus, more detailed analyses of participants’ attentional patterns while looking at the materials and thinking about them are needed to distinguish attentional benefits from costs in Quadrant 1.

## 4 CONCLUSIONS

Our results are largely consistent with basic assumptions about attention and learning, specifically that the time spent not thinking about the learning materials negatively affects learning. Thus, our results provide evidence for the validity of our categorization of learners’ attentional and cognitive states within the proposed 2×2 matrix.

There are two major challenges to characterizing learner’s complex attentional states during online learning, when using only eye tracking. 1) When the viewer is looking on screen, the eye tracker on its own cannot distinguish whether the viewer is thinking about what they are looking at. 2) Conversely, when the learner is looking away from the screen, that is all the eye tracker can only tell us. The first challenge is frequently overcome by measuring mind wandering using probes [Smallwood and Schooler, 2006, Weinstein, 2017], self-caught instances [Schooler et al., 2011], or automated measures using biometrics with machine learning [Hutt et al., 2019, Lee et al., 2022]. Tackling the second challenge is a key methodological contribution of our study. To go beyond what eye tracking can tell us, we distinguished between two cases in which the learner was *not* looking at the materials: those when the learner was on-task (Q2), versus off-task (Q4). Methodologically, there are three keys to our distinguishing these on-task versus off-task states while the learner was looking away from the materials: 1) using an egocentric camera to show what the learning was looking at; 2) using a retrospective recall interview to ask the learner to confirm what they were doing at that time; 3) having trained coders to do detailed coding for these states.

Nevertheless, an important limitation of our study is the minimal time participants spent in off-task behaviors, likely due to the demand characteristics of the lab study. Another possible explanation for our low times off-task is that our participants were giving socially desirable responses (i.e., saying what would make them look good), as shown previously for mind wandering study [Marcusson-Clavertz and Kjell, 2019]. We plan to address this in our follow-up studies, where we will collect data in the wild (e.g., home, coffee shop, etc.) and utilize attentional-aware machine learning software trained on our current ground-truth data to categorize learner’s attentional states using only webcam data.

There are two key purposes of our coding of these states during online learning. The first is to carry out important basic research on the relationship between our coded attentional and cognitive states and learning, using D’Mello’s [D’Mello, 2016] proposed 2×2 matrix. This matrix captures both information on whether the learner is looking at the materials versus elsewhere, and information on whether the learner is thinking about the learning content (i.e., onvs. off-task). To our knowledge, no prior studies have captured the effects of this full range of overt and covert attentional states on learning in a single study. Our initial results show that time spent in the four quadrants of the 2×2 matrix affects learning mostly as predicted. Our on-going basic research will investigate these research questions in greater depth.

The second purpose of our coding of these overt and covert attentional states is to develop applications for attention-aware intelligent-tutoring/online-learning. Critically, this requires the use of machine learning. Our detailed coding of the states in the 2×2 matrix provides ground truth data from the right hand column (looking elsewhere), which has been greatly facilitated by using egocentric cameras and retrospective recall interviews. Our on-going research is focused on leveraging this rich ground-truth data to extract additional features from our webcam data, which might not otherwise be extractable by machine learning.

Machine learning based on our ground truth data could be used in attention-aware educational software for estimating learners’ attentional and cognitive states in the 2×2 matrix for both supply side and demand side purposes. For the supply side, the software could show instructional designers the proportion of learners estimated to be off-task (not thinking about the learning materials), at each time point in an instructional module. Instructional designers can use such information to modify their modules in ways to better engage their students [Baker et al., 2020], for example switching from lecture to practical learning activities such as problem-solving tasks or case studies. For the demand side, whenever a learner’s focus on the material measurably drops, attention-aware software can prompt learners to briefly summarize what they just learned, then analyze that with a large language model for accuracy, and suggest reviewing that material if their estimated comprehension is below a threshold level [Mills et al., 2020]. Alternatively, if the learner is estimated to be sleepy, they can be asked if they would like to take a short break to refresh themselves and increase their learning [Ginns et al., 2023].

## CCS CONCEPTS

• Applied computing; • Education; • Computer-assisted instruction; • E-learning;
